# Phylum Level Change in the Cecal and Fecal Gut Communities of Rats Fed Diets Containing Different Fermentable Substrates Supports a Role for Nitrogen as a Factor Contributing to Community Structure

**DOI:** 10.3390/nu7053279

**Published:** 2015-05-06

**Authors:** Martin Kalmokoff, Jeff Franklin, Nicholas Petronella, Judy Green, Stephen P.J. Brooks

**Affiliations:** 1 Atlantic Food and Horticulture Research Station, Agriculture and Agri-Food Canada, Kentville, Nova Scotia B4N 1J5, Canada; E-Mails: Martin.Kalmokoff@agr.gc.ca (M.K.); Jeff.Franklin@agr.gc.ca (J.F.); 2 Bureau of Food Surveillance and Science Integration, Food Directorate, Tunney’s Pasture, Ottawa, Ontario K1A 0K9, Canada; E-Mail: Nicholas.Petronella@hc-sc.gc.ca; 3 Bureau of Nutrition, Food Directorate, Health Canada, Tunney’s Pasture, Ottawa, Ontario K1A 0K9, Canada; E-Mail: Judy.Green@hc-sc.gc.ca

**Keywords:** cecum, feces, bacterial community, metagenome, diet

## Abstract

Fermentation differs between the proximal and distal gut but little is known regarding how the bacterial communities differ or how they are influenced by diet. In order to investigate this, we compared community diversity in the cecum and feces of rats by 16S rRNA gene content and DNA shot gun metagenomics after feeding purified diets containing different fermentable substrates. Gut community composition was dependent on the source of fermentable substrate included in the diet. Cecal communities were dominated by *Firmicutes*, and contained a higher abundance of *Lachnospiraceae* compared to feces. In feces, community structure was shifted by varying degrees depending on diet towards the *Bacteroidetes*, although this change was not always evident from 16S rRNA gene data. Multi-dimensional scaling analysis (PCoA) comparing cecal and fecal metagenomes grouped by location within the gut rather than by diet, suggesting that factors in addition to substrate were important for community change in the distal gut. Differentially abundant genes in each environment supported this shift away from the *Firmicutes* in the cecum (e.g., motility) towards the *Bacteroidetes* in feces (e.g., *Bacteroidales* transposons). We suggest that this phylum level change reflects a shift to ammonia as the primary source of nitrogen used to support continued microbial growth in the distal gut.

## 1. Introduction

Diet has a major influence on the microbial community associated within the gut of mammals [1,2,3,4,5]. Dietary changes, including the addition of a single type of fermentable carbohydrate, can elicit a complex response as indicated by changes in the abundance of individual fecal species, changes to community species richness, community structure, the total bacterial load, and the collective metabolic activity of the gut bacteria [1,2,6,7]. For example, increases in the abundance of *Ruminococcus bromii* have been observed in the fecal communities of humans [1,8,9] and rodents [10,11] in response to diets containing type-2 resistant starch, as well as in the rumen of cattle fed barley containing rations [12]. Similarly, dietary fructans [13], galactooligosaccharides [14], isomalt [15], lactitol [16], and arabinogalactans [17], among other polymers have been associated with increases in the fecal load of Bifidobacteria in monogastric animals, although in the case of fructans, the effect is known to vary [18]. Diet can also mediate change in gut community structure at the phylum level. For example, in rodents fed high energy diets, a consistent observation has been the decrease in the *Bacteroidetes/Firmicutes* ratio in both feces and cecal contents, resulting in communities dominated by the *Firmicutes* [4,19]. Similar observations have been made in obese humans [20], although not all studies are supportive of this [21].

Diet mediated change is often viewed solely from the perspective of fermentable carbohydrate availability and most often by examining feces [22]. Gut bacteria do have nutritional requirements beyond fermentable carbohydrates, although the impacts of these additional requirements on gut community change are rarely considered. For example, many gut bacteria have a nutritional requirement for branch chain fatty acids (BCFAs), and their growth is therefore dependent on the end-products of the bacterial mediated gut protein fermentation [23]. Similarly, in gnotobiotic mice containing a defined model gut community and fed refined diets, casein content was the single factor which correlated with abundance for all species in this model system [24]. Recently, we concluded that community change in rats fed high amylose maize starch (type-2 resistant starch) was dependent on this substrate and ammonia derived from endogenous urea cycled into the proximal gut [11]. Together, these illustrate the point that fermentation is driven not only by available carbohydrate but can also be influenced by additional factors, such as nitrogen availability, required to sustain continued microbial growth [25].

Sources of fixed nitrogen available to support microbial growth in the gut also change depending on location (proximal gut *versus* the distal gut), although there is only a limited understanding of how this may impact change within the gut community. In the proximal colon, nitrogen is available primarily in the form of proteins, peptides and amino acids originating from the diet, and from various host factors such as sloughed off epithelial cells, mucin, and other digestive secretions [25]. The fermentation of these proteins, peptides and amino acids produces ammonia, a variety of secondary metabolites produced from various amino acids, and the BCFAs. Thus, ammonia concentrations in the colon are higher distally [26]. Since the nitrogen requirements of gut bacteria differ [27,28,29,30,31], it is not unreasonable to expect that those growing in the proximal gut may have different preferences for fixed nitrogen compared to those found in feces, and this might be reflected in changes in community composition or structure as well as differences in the fermentation [26,32]. In fact, this may be an important consideration when using dietary fermentable materials to influence factors such as butyrate production, since production is associated with specific gut lineages [33] whose activities may be localized to specific regions within the gut. While ammonia is often considered detrimental to the host, in the presence of fermentable carbohydrate, it can be used to support further microbial growth in the distal colon [34]. In fact, many species of gut bacteria do have a preference for ammonia as a fixed nitrogen source [28,31,35] and this preference could represent a selective factor enhancing their growth [11,23].

The above considerations predict a shift in the gut bacterial community from one dominated by species that utilize peptidyl nitrogen as a nitrogen source for growth in the proximal gut to one using ammonia in the distal gut. We tested this prediction by examining change in the cecal and fecal bacterial communities in rats fed purified diets containing different sources of fermentable carbohydrate.

## 2. Experimental Section

### 2.1. Feeding Trial

The feeding trial was carried out as previously described [2,11]. Briefly, 28 to 42-day old male BioBreeding control rats (*n* = 8/diet) were fed AIN93G purified diets [36] containing cellulose (C: 5% *w*/*w*), or wheat bran (WB: 5% *w*/*w*), or high amylose maize starch (RS: 5% *w*/*w* resistant starch content) over a 6-week period. Rats were individually housed in suspended wire bottom cages with ½ the floor covered by a solid metal plate, had free access to water, food and environmental enrichment, and were maintained on a 12 h light/dark cycle (21 °C and 40% humidity). Fecal pellets were collected from each rat at the termination of the feeding trial (day-28). Previous trials utilizing these substrates demonstrated that fecal community structure was stable within 7 days following the initiation of feeding these diets [2,11]. A balance trial was undertaken following the termination of the feeding trial with the rats maintained on these diets for an additional two weeks. Determinations carried out over the course of the balance phase included total feed consumption, total fecal outputs, fatty acid outputs, and residual substrate remaining in feces. Cecal contents were collected from each rat during necropsy following completion of the balance phase (day-42). This study was approved by the Health Canada Animal Care Committee.

### 2.2. SCFA and BCFA Analysis

Fecal and cecal short chain fatty acids (SCFA) and branch chain fatty acids (BCFA) concentrations were determined by capillary gas-liquid chromatography in samples collected from individual rats at the termination of the balance phase (day-42) as previously described [37]. 

### 2.3. Isolation of Community DNA

Feces (1.0 g/rat) and cecal contents (1.0 g/rat) from individual rats were pooled according to location (cecal or fecal) and diet (C, or WB or RS, *n* = 8 rats/diet, six pools of samples). Samples were pooled to minimize the effects of inter-rat gut community variability. Pooling is not expected to be a confounding factor because the microbial communities have been shown to stabilize after 7 days when fed similar diets [2,11]. Pooled samples were frozen in liquid nitrogen in a pre-chilled mortar and pestle and ground to a fine powder. Community DNA was isolated from the ground material using the Qiagen DNA Stool Isolation Kit, carried out according to the manufacturer’s protocol for difficult to lyse bacteria (Qiagen, Ontario). DNA was quantified then stored frozen at −20 °C.

### 2.4. Gut Community 16S rRNA Gene Analysis

Bacterial tag-encoded FLX amplicon sequencing (454 Life Sciences, Roche, Branford, CT, USA) of fecal and cecal community 16S rDNA genes was carried out at Research Testing Laboratory (Lubbock, Texas, USA) using the forward primer F44 and reverse primer 519R encompassing the V1-2 region (bases 27–519) of the *Escherichia coli* 16S rRNA gene [11]. Read data files (~10,000 reads per file) were randomly sampled (3000 sequences) and the sequencing tag and primer removed using Seaview [38]. Phylotypes were initially binned using Esprit [39] at a cut-off resulting in ~200 operational taxonomic units (OTUs) per sample. These sequences were then aligned against the Silva database [40] and further binned according to their best match template with the most abundant sequence defined as the representative OTU. Additional binning of phylotypes was carried out by generating Neighbor joining trees and combining OTUs falling within a 3% sequence divergence cut-off. Each data set was checked for chimeric sequences using ChimeraCheck, as implemented through Mothur [41], with suspected sequences removed from each data set. Phylotypes which occurred less than 3 times from each data set or exhibited <0.80 similarity score with existing 16S rRNA sequences in the Ribosomal Data-base (RDP; http://rdp.cme.msu.edu) were also removed from each data set. Phylotypes were classified using the RDP [42]. 

### 2.5. Metagenomic Analysis

DNA Shot-gun metagenomic library construction and sequencing was carried out using cecal and fecal community DNA using standard protocols (454 Life Sciences, Roche, Branford, CT, USA). Roche GS-FLX Titanium sequencing was carried out at the Genome Quebec facility (Montreal, Canada). All six libraries were sequenced on a half plate. Initially, the raw reads were filtered for quality (minimum length of 40 base pairs, and a Phred quality score of at least 20), the linkers removed and the sequences subjected to a BlastX using the default parameters [43] against the all non-redundant GenBank CDS database to obtain community sequences, yielding libraries consisting on average of 112,344 ± 22,545 sequences. Sequences were analyzed using MEGAN [44], and both taxonomic profiles and assignments to the SEED database [45] within each community determined. Contiguous DNA sequences were assembled using the aforementioned filtered raw reads via VelvetOptimizer using the default parameters [46]. Contiguous sequences were also searched using BlastX against the CAZy database [47]. Meta-libraries for all six communities are deposited on the MG-RAST database under the following accession numbers: 4530763.3–4530768.3.

### 2.6. Statistical Analysis

Multidimensional scaling analysis (MDS) using principal co-ordinate analysis (PCoA) was carried out using PC-ORD software package [48]. 16S rRNA data matrices consisting of phylotype occurrence and abundance for each diet and location were used to generate ordination plots (PCoA) using Sorenson distances [49] with 1000 randomizations. Cluster analysis was carried out using a Sorenson distance measure and nearest neighbor linkage method. Diversity indices for 16S rRNA community data was calculated using FastGroup II on-line software [50]. 

For the analysis of metagenomic data, gene assignments to the SEED database [45] obtained from MEGAN were first organized into abundance matrices and assignments within each community were normalized (% of total). Normalized data in each community was highly skewed and sparse. In order to reduce the skew and scarcity across the data, we removed the least abundant assignments across all six communities (bottom 5%). Multidimensional scaling analysis (PCoA) was carried out using PC-ORD and clustered using Euclidian distances with 1000 randomizations. Groups within the ordination space were tested for significance using a Multi-Response Permutation Procedures (MRPP) implemented in PC-ORD. Cluster analyses of SEED data were calculated using Euclidean distances and Ward’s group linkage method using PC-ORD. To visualize trends in the data, de-trended correspondence analysis was performed on the normalized abundance data using the vegan package in R [51]. Scores from the first two components of the diet*location combinations and individual gene abundances were used to produce bi-plots. 

## 3. Results

### 3.1. Fecal and Cecal Community Metabolic Activity

Cecal and fecal community metabolic activity under each diet was assessed from SCFA profiles (Table 1). For all three diets, fatty acid concentrations were higher in the cecum compared to feces (data not reported) and total fecal SCFA and BCFA output (μmol·gdw^−1^·day^−1^) was approximately two-fold higher in rats fed WB or RS compared to those fed C. Cecal butyrate concentration as a proportion of total SCFA was two-fold higher in rats fed WB compared to those fed C or RS, although this was not the case in the feces, where the proportion of butyrate was similar across diets. The proportion of SCFA as propionate was higher in the cecum of rats fed C or RS compared to those fed WB, and remained significantly higher in the feces of those fed RS. Finally, appreciable levels of BCFAs occurred in both the cecum and feces of rats ingesting all three diets. BCFA concentrations in the feces of those fed WB or RS were higher than those fed C, although this difference was not significant.

**Table 1:** Short chain fatty acid (SCFA) and branch chain fatty acid (BCFA) concentrations expressed as a percentage of the total in feces and cecal contents, and daily fecal outputs (μmol·gdw^−1^·day^−1^) under each diet. Values represent mean ± SEM (*n* = 5–8), those with different superscripts (a or b) are significantly different as determined by Tukey HSD at the *p* < 0.05 level.

Fatty Acid	Cecal (%)			Fecal (%)			Fecal (μmol·gdw^−1^·y^−1^)		
	C	WB	RS	C	WB	RS	C	WB	RS
Acetic	59.4 ± 0.6	53.7 ± 1.1	59.9 ± 0.8	73.0 ± 1.0 ^a^	72.6 ± 1.1 ^b^	57.5 ± 0.9 ^b^	48.0 ± 3.4 ^a^	103.5 ± 4.5 ^b^	79.7 ± 2.4 ^b^
Propionic	19.1 ± 0.2 ^a^	14.6 ± 0.3 ^b^	20.3 ± 0.6 ^a^	10.7 ± 0.2 ^a^	11.7 ± 0.6 ^a^	21.6 ± 0.9 ^b^	6.9 ± 0.5 ^a^	16.4 ± 0.8 ^b^	30.3 ± 1.7 ^b^
Butyric	12.8 ± 0.2 ^a^	25.3 ± 0.8 ^b^	11.2 ± 0.4 ^a^	8.2 ± 1.2	8.2 ± 0.8	10.8 ± 0.4	5.2 ± 1.0 ^a^	13.1 ± 1.8 ^b^	14.8 ± 0.6 ^b^
Isobutyric	2.3 ± 0.1 ^a^	1.7 ± 0.02 ^b^	1.8 ± 0.04 ^ab^	1.4 ± 0.2 ^a^	1.7 ± 0.1 ^b^	1.6 ± 0.0 ^ab^	1.0 ± 0.2	2.3 ± 0.2	2.2 ± 0.1
Isovaleric	3.0 ± 0.1 ^a^	2.0 ± 0.02 ^b^	1.8 ± 0.06 ^b^	3.4 ± 0.3	2.7 ± 0.2	2.8 ± 0.1	2.3 ± 0.2	3.9 ± 0.3	3.8 ± 0.1
Valeric	3.4 ± 0.1	2.6 ± 0.04	2.9 ± 0.1	2.5 ± 0.3 ^a^	2.5 ± 0.1 ^a^	3.9 ± 0.1 ^b^	1.6 ± 0.2	3.5 ± 0.3	5.4 ± 0.2
Caproic	0.03 ± 0.01	0.13 ± 0.05	1.4 ± 0.2	0.7 ± 0.2 ^a^	0.6 ± 0.1 ^a^	1.3 ± 0.1 ^b^	0.4 ± 0.1	1.0 ± 0.2	1.9 ± 0.2
Heptanoic	0.0 ± 0.0	0.0 ± 0.0	0.6 ± 0.10	0.0 ± 0.0	0.0 ± 0.0	0.5 ± 0.1	0.0 ± 0.0	0.0 ± 0.0	0.7 ± 0.1
Total	-	-	-	-	-	-	65.5 ± 4.6 ^a^	143.7 ± 6.9 ^b^	138.9 ± 3.8 ^b^

### 3.2. 16S rRNA Profiling of Fecal and Cecal Communities

Cecal and fecal community composition was initially determined by analyzing 16S rRNA gene content generated through pyrosequencing. In total, 155 phylotypes were identified across all six communities, although only 13 of these (8%) were shared (Supplemental Table 1). Phylotype richness (Chao1) varied with diet and location (Table 2). Fecal and cecal contents from rats fed WB or C were richer than those fed RS, and diversity (Shannon index) within the RS-fed cecal and fecal communities was unevenly distributed (Table 2). A higher proportion of phylotypes were shared between each respective cecal and fecal community (~60%–70%), than found in comparisons between all three cecal or all three fecal communities (~25%–40%).

**Table 2:** Community richness (Chao1) and diversity (Shannon index) for cecal and fecal samples from rats fed each diet, calculated using FAST Group II software [50].

Index	Cecal			Fecal		
Community	C	WB	RS	C	WB	RS
Chao1	122	131	83	111	153	93
Shannon index	3.6	3.8	2.5	3.5	3.8	2.5

**Figure 1:**
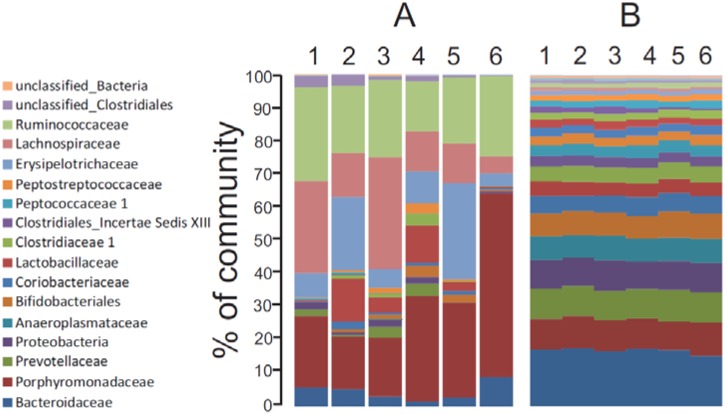
(**A**) Distribution of phylotypes (% of total) at the family taxon level in the fecal and cecal communities of rats fed each diet. Family taxa are identified on the left of panel A. The phylum *Proteobacteria* encompasses the families *Desulfovibrionaceae*, *Helicobacteraceae*, *Sutterellaceae*, *Pasteurellaceae* and unclassified “*Proteobacteria*”; (**B**) Distribution of gene assignments among the 26 identified SEED families (% of total). SEED families in ascending order are Carbohydrates, Virulence, Protein Metabolism, DNA Metabolism, Cell Wall and Capsule, Amino Acids and Derivatives, RNA Metabolism, Clustering Based Subsystems, Cofactors and Vitamins, Cell Division and Cell Cycle, Nucleosides and Nucleotides, Respiration, Stress Response, Regulation and Cell Signaling, Fatty Acids, Motility and Chemotaxis, Phages and Transposons, Membrane Transport, Phosphorus Metabolism, Dormancy and Sporulation, Nitrogen Metabolism, Miscellaneous, Sulfur Metabolism, Metabolism of Aromatic Compounds, Secondary Metabolism, and Potassium Metabolism. Lane 1: C cecal community. Lane 2: C fecal community. Lane 3: WB cecal community. Lane 4: WB fecal community. Lane 5: RS cecal community. Lane 6: RS fecal community.

The distribution of phylotypes at the family level within each community under each diet (Figure 1A) showed a number of trends. First, the majority of phylotypes aligned into four dominant families including the *Porphyromonadaceae*, *Erysipelotrichaceae*, *Lachnospiraceae*, and *Ruminococcaceae* despite differences in location or diet. Second, *Porphyromonaceae* was the dominant family in the phylum *Bacteroidetes* under all diets, and the dominant lineage in the feces of rats fed RS. Third, the cecal communities contained a higher content of *Lachnospiraceae* than found in each respective fecal community. Finally, the family *Lactobacillaceae* was a major taxon only in the fecal communities of rats fed the C or WB-supplemented diets.

**Figure 2:**
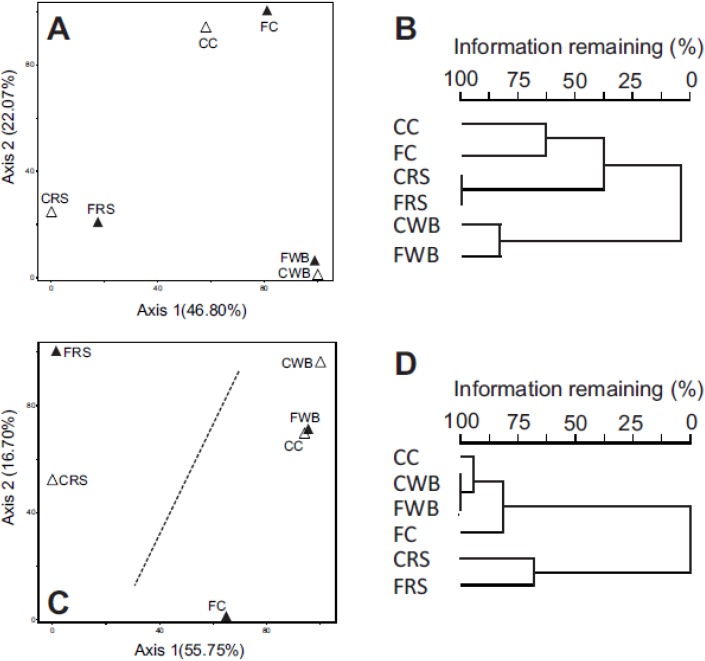
Multi-dimensional scaling (MDS) analysis using principle coordinates (PCoA) and cluster analysis of phylotype composition (presence/absence) and diversity (phylotype presence and abundance) in the cecal (Δ) and fecal (▲) communities of rats fed each diet. (**A**) MDS comparing phylotype content under each diet. *p* = 0.001, *r*^2^ = 0.813; (**B**) Cluster analysis comparing phylotype content under each diet; (**C**) MDS comparing diversity under each diet. *p* = 0.001, *r*^2^ = 0.960; (**D**) Cluster analysis comparing diversity under each diet. CC: cecal cellulose. FC: fecal cellulose. CWB: cecal wheat bran. FWB: fecal wheat bran. CRS: cecal resistant starch. FRS: fecal resistant starch.

Multi-dimension scaling analysis (MDS) by principal co-ordinate analysis (PCoA) comparing phylotype composition (presence/absence) across all six communities, grouped according to diet (Figure 2A), indicating that the fermentable substrate contained within each diet defined gut community composition. A cluster analysis yielded an identical result (Figure 2B). In contrast, comparisons of community diversity (phylotype occurrence and abundance) no longer partitioned exclusively on diet (Figure 2C). Firstly, respective cecal and fecal communities under each diet were not as closely related as found in comparisons of community composition. Secondly, the cecal community in rats fed C was more similar to the WB communities, rather than to its corresponding fecal community. The six communities fell within two main groups within the ordination space, with the RS fecal and RS cecal communities grouping separately. This grouping was significant (MRPP; *p* = 0.018) and was also supported by a cluster analysis (Figure 2D) suggesting that other factors in addition to fermentable substrate were also contributing to diversity within these communities.

### 3.3. Taxonomic Profiling by Analysis of Community Shot Gun DNA Sequences

Cecal and fecal community structure was also determined based on the assignment of sequence reads to the lowest common ancestor using MEGAN [44]. Reads assigned to the kingdoms Bacteria and Archeae (18,653 ± 2963/library) represented 27.1% ± 2.4% of the total reads within each respective library. MEGAN assigned a larger proportion of these bacterial DNA sequences to the phylum *Bacteroidetes* than indicated by the 16S rRNA gene analysis. A comparison of the *Bacteroidetes* content among all six communities is presented in Table 3. We also checked sequence assignments at the phylum level using MG-RAST [52] and for contiguous DNA sequence hits from the carbohydrate-active enzymes database [47]. While there was a degree of variability among the different approaches, all three indicated a trend towards increased *Bacteroidetes* content across the cecal communities and in the fecal communities from rats fed C or WB. Estimates for *Bacteroidetes* content in the fecal community of rats fed RS were slightly lower on average compared to that based on the analysis of 16S rRNA genes.

**Table 3:** *Bacteroidetes* content (% of total) in cecal contents and feces under each diet as determined by the assignment of 16S rRNA genes or by assignment of DNA sequences (MEGAN, MG-RAST) or contiguous (CAZymes) DNA sequences.

Estimator	Cecal Contents			Feces		
	C	WB	RS	C	WB	RS
16S rRNA ^1^	29	24	31	22	37	64
MEGAN	36	31	46	43	56	64
MG-RAST	28	24	39	34	41	56
CAZymes	33	27	39	40	53	53
Average ^2^	32	27	41	39	50	58

^1^ RDP using a 0.95% confidence interval; ^2^ Average of determinations involving the assignment of randomly cloned DNA (MEGAN, MG-RAST, and CAZymes).

Phylogenetic assignments below the phylum level were not consistent with results from 16S rRNA sequence analysis. For example phylotypes in the family *Erysipelotrichaceae* accounted for 6%–29% of total 16S rRNA reads across all six communities, but only 1%–5% of gene assignments at the family level. Similarly in the phylum *Bacteroidetes*, MEGAN assigned the majority of reads to the family *Bacteroidaceae*; whereas the 16S rRNA analysis indicted that the family *Porphyromonadaceae* was the dominant lineage within the *Bacteroidetes*. This discordance likely reflects the paucity of full length genomic sequences available for isolates from the rat gut community. 

### 3.4. Metagenome Profiling by Analysis of Community DNA Sequences

**Figure 3:**
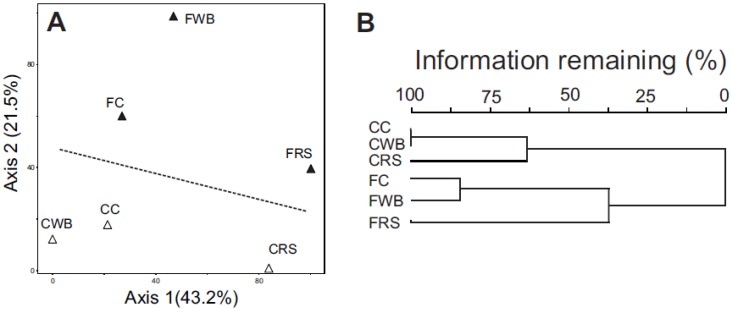
(**A**) MDS (PCoA) analysis of SEED gene assignments in the cecal (Δ) and fecal (▲) communities of rats fed each diet. Ordination plot was calculated using Euclidian distances and with resampling 1000 times. Axis 1: 43.2% of data variability, *R*^2^ = 0.746, *p* = 0.0009. Axis 2: 21.5% of data variability, *R*^2^ = 0.848, *p* = 0.0009; (**B**) Cluster analysis of SEED gene assignments. CC: cecal cellulose. FC: fecal cellulose. CWB: cecal wheat bran. FWB: fecal wheat bran. CRS: cecal resistant starch. FRS: fecal resistant starch.

Functional gene profiles across the identified SEED categories were similar among all six communities (Figure 1B), despite the differences in community composition and structure (Figure 1A, Table 3). Similar to other gut metagenomes [53,54,55], genes involved in the metabolism of carbohydrates accounted for the largest proportion of SEED hits (16% of total), with the top five categories (Carbohydrates, Virulence, Protein Metabolism, DNA Metabolism, and Amino Acids and Derivatives) encompassing >50% of the assignments within each community. Despite the overt similarity in gene content across all six communities, MDS analysis (PCoA) comparing SEED gene assignments across all six communities formed into distinct groups (Figure 3A). Firstly, there was a separation between the cecal and fecal environments under all three diets with the cecal communities occupying the lower region of the ordination space and the fecal communities the upper region. Secondly, the cecal and fecal communities in rats fed RS appeared to be more closely related to each other than to the other communities. However, cluster analysis also indicated the all six communities clustered by environment (Figure 3B) and this grouping was statistically significant (MRPP: *p* = 0.031), whereas clustering of the RS communities as a separate group was not (*p* = 0.286).

**Figure 4:**
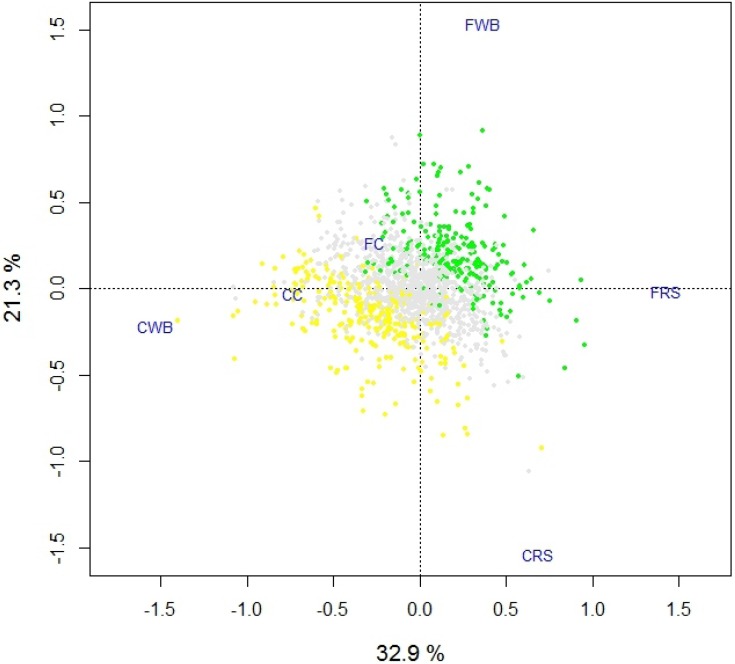
Biplot (PCA) showing the ordinal location for each SEED gene assignment (Supplementary Table 2) and their relationship to each community. SEED gene assignments having >50% increase in abundance in the fecal or cecal communities are indicated (green and yellow points respectively), all others are indicated in grey. CC: cecal cellulose. FC: fecal cellulose. CWB: cecal wheat bran. FWB: fecal wheat bran. CRS: cecal resistant starch. FRS: fecal resistant starch.

In order to understand the factors contributing to the partitioning of the cecal and fecal communities, we identified SEED gene assignments having an increased abundance in both the cecal and fecal communities. Of 1449 total SEED gene assignments (Supplementary Table 2), 294 assignments in the ceca and 257 in the feces were identified as having >50% increase in abundance. These differentially abundant gene assignments clustered furthest from the ordinal center of a PCA plot and appear to drive the partitioning by environment (Figure 4). Many of these differentially abundant genes were reflective of the shift from environments dominated by the *Firmicutes* (ceca) to those containing an increased content of *Bacteroidetes* (feces). For example, cecal communities were enriched in SEED categories, subcategories and genes involved in Motility and Chemotaxis; Dormancy and Sporulation; Cell wall and Capsule (capsular and extracellular polysaccharides, teichoic acids); Virulence (Type III, Type IV, Type VI, ESAT secretion systems, proteins containing the LPXTG cell wall binding motifs). In fact, the majority of gene assignments in the motility and chemotaxis SEED category (78%) were to the family *Lachnospiraceae*, and many of the genera in this family are motile [56,57]. In contrast, the fecal communities were enriched in SEED categories, subcategories and genes involved in Amino acid and Derivatives (ammonia assimilation, urea cycle and polyamines); Cofactors, Vitamins and Prosthetic Groups (coenzyme B12 biosynthesis, menaquinone and phylloquinone biosynthesis); Phages, Prophages, and Transposable Elements (*Bacteroidales* conjugative transposons); Protein Metabolism (protein degradation, stress response, rubrerythrin); and Virulence (Ton and Tol transport systems).

### 3.5. Potential Community Glycoside Hydrolase Capacity

Contiguous DNA sequences generated from each community were searched against the CAZY database [47] in order to examine the potential glucoside hydrolase capacity within each environment. In total, 84 glycoside hydrolase families (GH), 49 carbohydrate binding motifs (CBM), 35 glycosyl transferases (GT), 12 carbohydrate esterases (CE), 8 polysaccharide lyases and 5 auxiliary activities (AA) were identified across all six communities (Supplemental Table 3). Results from ordination and cluster analysis were similar to that found in comparisons of community diversity (Figure 2A), in that the RS fecal and cecal community were more closely related but quite different from the other communities and this grouping was significant (MRPP; *p* = 0.023). Highly abundant (>1% of total) glycosyl-hydrolase families and their associated CBMs occurring in each environments are listed in Table 4. Glycosyl hydrolases and their associated CBMs encompassed ~60% of the CAZymes identified within each environment. The most abundant GH families were involved in the metabolism of starch (GH13, GH13|CBM26, GH77, and GH77|CBM20), enzymes involved in the metabolism of cellulose and hemicellulose in plant cell walls (GH3, and GH43), lysozyme (GH23), and enzymes for cleavage of β-galactosides such as those occurring in mucin (GH2).

## 4. Discussion

Feeding purified diets containing different substrates yielded cecal and fecal communities which were quite different from one another in terms of phylotype composition, community structure, and collective metabolic activities. Cellulose (C) is generally included in rodent diets as a non-digestible bulking agent [36]. However, the substrate does undergo a limited fermentation (8% *w*/*w* utilized) yielding two-fold lower fecal SCFA outputs than the other two diets. Wheat bran (WB) is a more complex substrate and contains hemicellulose (xylan and arabinoxylan), starch, fructan, covalently bound protein and cellulose [58,59]. In rats, the fermentation of WB occurs largely at the expense of hemicellulose [59] and is relatively slow with appreciable quantities being excreted in the feces (47% *w*/*w* utilized). Fermentative end-product profiles produced from either substrate differed in the cecum where WB was butyrogenic and C propiogenic, but was very similar in the feces where acetate was the dominant SCFA at the expense of both butyrate and propionate. In contrast to C and WB, high amylose maize starch (RS) is fermented to completion, and is associated with relatively higher propionate concentrations in both the cecum and feces. The cecal and fecal communities in rats fed RS also differed from the other communities in being less rich in terms of OTU content, and contained an uneven distribution of phylotypes. Both features result from an overgrowth of the gut community by several yet to be cultivated species in the family *Porphyromonadaceae* and by *Ruminococcus bromii*. Gut community composition and structure under this diet is partially driven by ammonia derived from endogenous urea cycled into the cecum [11]. 

**Table 4:** Abundant glycosyl hydrolase families (>1% of total abundance in at least one community) identified in the cecal and fecal communities of rats fed each diet. CC: cecal cellulose. FC: fecal cellulose. CWB: cecal wheat bran. FWB: fecal wheat bran. CRS: cecal resistant starch. FRS: fecal resistant starch.

CAZyme Family	Function	% Abundance					
		CC	CWB	CRS	FC	FWB	FRS
GH3	β-glucosidase, xylan 1,4-β-xylosidase, glucan 1,3-β-glucosidase, *etc.*	6.0	4.5	3.9	7.8	6.0	5.9
GH2	β-galactosidase, β-glucuronidase, β-mannosidase, *etc.*	5.2	4.6	3.6	5.8	4.7	3.2
GH13	α-amylase, pullulanase, *etc.*	4.4	3.9	5.4	2.0	3.4	5.4
GH23	lysozyme type G, peptidoglycan lyase, chitinase.	3.9	2.9	3.6	5.0	4.5	2.7
GH43	β-xylosidase, β-1,3-xylosidase, α-l-arabinofuranosidase, arabinanase, xylanase, *etc.*	3.5	2.5	2.7	1.8	3.5	2.0
GH31	α-glucosidase, α-1,3-glucosidase, α-xylosidase, *etc.*	2.3	1.9	2.9	1.3	1.7	1.2
GH13|CBM26	GH13 + starch binding module.	1.1	0.4	3.1	0.3	1.3	3.7
GH92	α-mannosidase, α-1,2-Mannosidase, *etc.*	1.8	0.4	1.0	1.4	1.3	1.7
GH36	α-galactosidase, α-N-acetylgalactosaminidase, stachyose synthase, raffinose synthase	0.4	1.8	1.2	1.1	1.9	1.0
GH73	peptidoglycan hydrolase with endo-β-N-acetylglucosaminidase specificity.	0.5	1.1	0.7	2.0	1.7	1.0
GH97	α-glucosidase, α-galactosidase.	0.4	0.6	1.7	0.8	2.0	1.6
GH94	cellobiose phosphorylase, cellodextrin phosphorylase, chitobiose phosphorylase, *etc.*	1.0	1.1	1.1	1.8	0.9	0.7
GH1	β-glucosidase , β-galactosidase, 6-P-β-glucosidase, β-glucuronidase, β-d-fucosidase, *etc.*	1.2	0.8	0.5	2.5	0.6	0.2
GH32	invertase, endo-inulinase, endo-levanase, exo-inulinase, *etc.*	1.2	1.4	0.0	1.3	0.8	0.8
GH77	amylomaltase or 4-α-glucanotransferase.	1.0	1.3	1.2	0.6	0.2	0.5
GH51	α-l-arabinofuranosidase, endoglucanase	0.9	0.9	1.0	0.5	0.5	0.9
GH95	α-1,2-l-fucosidase, α-l-fucosidase	0.4	0.1	0.5	1.6	0.7	1.0
GH20	β-hexosaminidase, lacto-N-biosidase, β-1,6-N-acetylglucosaminidase, *etc.*	0.7	0.6	0.2	1.2	1.1	0.5
GH29	α-l-fucosidase, α-1,3/1,4-l-fucosidase	0.7	1.0	0.6	0.6	0.5	0.7
GH25	lysozyme	1.1	1.0	0.4	0.4	0.5	0.8
GH39	α-l-iduronidase, β-xylosidase	0.5	0.8	0.6	0.8	0.5	0.6
GH18	chitinase, lysozyme, endo-β-N-acetylglucosaminidase, peptidoglycan hydrolase, *etc.*	0.9	0.4	0.5	0.6	0.6	0.8
GH105	unsaturated rhamnogalacturonyl hydrolase.	0.8	0.1	0.6	0.8	0.5	0.8
GH127	β-l-arabinofuranosidase.	1.5	0.0	0.4	0.6	0.8	0.2
GH2|CBM32	GH2 + binding to galactose, lactose, polygalacturonic acid, *etc.*	0.0	0.1	0.1	0.9	1.4	0.8
GH77|CBM20	GH77 + The granular starch-binding function.	0.1	0.6	0.7	0.5	0.6	0.7

Comparisons of phylotype composition between all six communities indicated that the cecal and fecal communities grouped by diet, demonstrating that the fermentable substrate within each diet defined community composition (Figure 1A). While phylotype composition remained similar between the cecum and feces under each diet, the abundance of individual phylotypes did change and this impacted community structure to varying degrees depending on the diet. For example, the relative abundance of phylotypes aligning within the family *Lachnospiraceae* were higher in the ceca compared to the feces irrespective of the diet, although the actual content (% of total) in each cecal and fecal community was quite different between diets. Changes in phylotype abundance not only altered community structure, but also how the six communities related to one another (Figure 1B). Moreover, these changes occurred independently of the fermentable substrate, suggesting that additional yet to be identified factors also contribute towards the community changes occurring between the cecum and feces under each diet. 

Community structure determined by the assignment of individual DNA sequences (MEGAN, MG-RAST) or contiguous DNA sequences (CAZY) to the phylogeny yielded results which were different from those based on 16S rRNA genes (Table 3). While community profiling based on 16S rRNA gene content can provide a reasonable overview of a microbial community, the approach does suffer certain limitations [60]. Firstly, ribosomal gene copy numbers do vary across the bacterial kingdom. For example, the average copy number for *Bacteroidetes* is 3.4 compared to 6.8 for the *Firmicutes* [61]. Not only is it difficult to assess the degree that copy number may impact the inferred community structure, it is also very difficult to correct for since very few rat bacterial genomes have been sequenced. Secondly, pyrosequencing requires an initial PCR step (25 cycles of amplification), which can potentially contribute additional artifacts which may have an impact on the community analysis [60]. Alternative approaches to minimize artifacts in gene based community analyses include building phylogenies based on single copy genes [62], or through the use of shot gun metagenomics to describe community structure [63,64]. We suspect that 16S rRNA analyses may be confounded by differential 16S rRNA copy numbers, and this was most evident in communities containing a higher proportion of *Firmicutes*. For example, in rats fed the C or WB containing diets we found a considerable difference in estimates of the *Bacteroidetes* content in feces between the 16S rRNA gene analysis and that based on the assignment of randomly sequenced clones. Moreover, community structure based on the assignment of random sequences supported a shift of varying degrees towards the *Bacteroidetes* in all three fecal communities, which was not always apparent in the 16S rRNA gene analyses. In contrast to the fecal communities, the cecal communities consisted primarily of *Firmicutes*, with the families *Lachnospiraceae*, *Ruminococcaceae* and *Erysipelotrichaceae* encompassing the majority of the phylotype abundance in this portion of each community. 

In comparisons of gene content, each cecal and fecal community grouped by environment rather than by diet (Figure 3), which was quite surprising considering the differences in community composition and structure between the three cecal and three fecal communities, and the differences in the nature and degree of fermentability of each substrate provided in the diet. Based on our examination of differentially abundant genes (Figure 4), it is also clear that the shift away from the *Firmicutes* in the cecum towards the *Bacteroidetes* in the feces observed under all three diets is likely responsible for this relationship. The important question concerns the significance of this change in community structure. 

Community shifts towards the *Bacteroides* have previously been observed in continuous human fecal cultures in response to increases in pH [65]. In fact, the authors suggested that the mildly acidic conditions occurring in the proximal gut of humans, might limit the growth of *Bacteroides* populations at this site. However, we do not think that this situation is directly comparable to that occurring in our rats. Firstly, both the cecum and feces in rats fed C are slightly basic (pH = 8.3 and 7.5 respectively), and even when fed a substrate which is completely fermented, like RS. For example, the pH at either location remains in the slightly basic to neutral range (pH = 7.7 and 7.0 respectively) [11]. Secondly, *Bacteroidetes* abundance appears to be related to additional urea derived ammonia in the cecum associated with RS feeding [11].

While it has long been known that the availability of different forms of nitrogen changes between the proximal and distal gut [26,32] there is little information regarding the extent to which this might impact gut communities. Peptidyl nitrogen originating from the diet and a variety of endogenous sources are readily available in the proximal gut [32]. Since appreciable quantities of BCFAs occurred in the cecum under each diet, it appears that protein fermentation occurs concurrently with carbohydrate fermentation so that ammonia would also be available to support microbial growth. While the nitrogen requirements of bacteria in the proximal gut have not been well characterized, a previous study examining nitrogen requirements among isolates from the cecum of horses did find that most utilized peptone as the sole nitrogen source, while much fewer were also able to utilize either ammonia or urea [30]. Dominance of the *Firmicutes* in the cecum of these rats could reflect a preference for peptidyl-nitrogen as nitrogen source [30], or arise from the stimulatory effect of peptidyl-nitrogen on bacterial growth as has previously been observed in ruminants [66], or merely reflect a greater flexibility in terms of nitrogen requirements among the *Firmicutes* compared to the *Bacteroidetes*.

Ammonia levels increase as you move distally down the gut, reflecting the continued fermentation of proteins, peptides and amino acids [26,32]. In the presence of fermentable carbohydrate, ammonia will be used to support the continued bacterial growth [34]. Similar to rumen bacteria, bacteria isolated from the feces of monogastric animals are generally believed to utilize ammonia as their primary nitrogen source [67]. Indeed, most species in the *Bacteroides* [31] and *Prevotella* [35,68] do have a preference for ammonia, whereas others species such as *Ruminococcus bromii* have an absolute requirement [23]. On these bases, it is not unreasonable to suggest that the observed shift in these gut communities towards the *Bacteroidetes* likely follows from increases in the concentration of gut ammonia as you move distally. The primary difference between the fecal communities of rats fed any of these diets is the extent of this overgrowth by the *Bacteroidetes*. Conversely, under conditions where appreciable urea is not cycled into the cecum (feeding the C or WB diets) growth of species in the cecum having a preference for ammonia might ultimately depend on the rate of protein fermentation.

An important question is whether these findings are applicable to humans. First, rat feeding trials are highly controlled where a constant diet is fed over an extended period, a situation which cannot be reproduced in human trials. Protein availability in these refined diets is also very different from a typical human diet since the primary dietary protein source is casein. Obviously, diets containing multiple protein sources of differing digestibility may affect nitrogen availability in more complex ways. Unfortunately, there is little to no information regarding diet mediated change along the human gut tract, or in other monogastic animals, with most of the previous analyses on diet mediated change utilizing feces. However, we do know that the genus *Bacteroides* is the dominant lineage in the human feces [69,70], so it is quite possible that the changes observed in rats represent a common change within the gut communities of monogastic animals. From a practical perspective, our results suggest that it is important to consider the effects of available nitrogen when using diet to mediate specific changes in the gut community, rather than fermentable carbohydrates alone. Similarly, given the involvement of taxa like the family *Lachnospiraceae* in butyrate production [71] and the potential role of butyrate in gut health [72], changes in abundance affected by location and possibly by the availability of peptidyl-nitrogen, illustrate potential difficulties in finding practical solutions for increasing butyrate concentration in the distal colon. Finally, it is of interest to note that a commonly observed change in the gut community of obese rodents fed high energy diets has been a shift in the gut community towards the phylum *Firmicutes* [4,19], and various explanations have been forwarded to explain this observation [73,74]. However, since the obese state in rats is also characterized by gut inflammation and alterations to barrier function [75], we suggest that gut leakage could have a significant impact on peptidyl-nitrogen availability, yielding cecal communities enriched in *Firmicutes* and fecal communities more similar in terms of composition and structure to those found in the cecum. 

## 5. Conclusions

Diet mediated change in the gut bacterial communities in monogastric animals are generally viewed exclusively from the perspective of fermentable carbohydrate availability. Here, we have presented evidence demonstrating that additional factors beyond strictly the source of fermentable carbohydrate contained in a diet are important in mediating community change. By examining community change in proximal (cecum) and distal (feces) communities from rats fed purified diets containing different sources of fermentable carbohydrate, we have shown that while community composition was dependent on the source of fermentable carbohydrate included in the diet, community structure was not. Cecal communities were dominated by the phylum *Firmicutes*, whereas in feces the community structure was shifted by varying degrees depending on the diet towards the phylum *Bacteroidetes*. This shared phylum level change can be explained on the basis of a shift from a community using peptidyl-nitrogen as the primary nitrogen source to support continued growth (proximal gut), to one using ammonia (distal gut). 

## Supplementary Files

Supplementary File 1
